# Phase stability and dense polymorph of the BaCa(CO_3_)_2_ barytocalcite carbonate

**DOI:** 10.1038/s41598-022-11301-w

**Published:** 2022-05-06

**Authors:** R. Chuliá-Jordán, D. Santamaría-Pérez, J. González-Platas, A. Otero-de-la-Roza, J. Ruiz-Fuertes, C. Popescu

**Affiliations:** 1 MALTA Consolider Team, Departamento de Física Aplicada-ICMUV, Universitat de València, 46100 Valencia, Spain; 2 MALTA Consolider Team, Departamento de Física, Instituto Universitario de Estudios Avanzados en Física Atómica, Molecular y Fotónica (IUDEA), Universidad de la Laguna, Avenida Astrofísico Fco. Sánchez S/N, La Laguna, 38204 Tenerife, Spain; 3 MALTA Consolider Team, Departamento de Química Física y Analítica, Facultad de Química, Universidad de Oviedo, 33006 Oviedo, Spain; 4 MALTA Consolider Team, DCITIMAC, Universidad de Cantabria, 39005 Santander, Spain; 5 CELLS-ALBA Synchrotron Light Facility, Cerdanyola del Vallès, 08290 Barcelona, Spain

**Keywords:** Solid Earth sciences, Astronomy and planetary science, Materials science

## Abstract

The double carbonate BaCa(CO_3_)_2_ holds potential as host compound for carbon in the Earth’s crust and mantle. Here, we report the crystal structure determination of a high-pressure BaCa(CO_3_)_2_ phase characterized by single-crystal X-ray diffraction. This phase, named post-barytocalcite, was obtained at 5.7 GPa and can be described by a monoclinic *Pm* space group. The barytocalcite to post-baritocalcite phase transition involves a significant discontinuous 1.4% decrease of the unit-cell volume, and the increase of the coordination number of 1/4 and 1/2 of the Ba and Ca atoms, respectively. High-pressure powder X-ray diffraction measurements at room- and high-temperatures using synchrotron radiation and DFT calculations yield the thermal expansion of barytocalcite and, together with single-crystal data, the compressibility and anisotropy of both the low- and high-pressure phases. The calculated enthalpy differences between different BaCa(CO_3_)_2_ polymorphs confirm that barytocalcite is the thermodynamically stable phase at ambient conditions and that it undergoes the phase transition to the experimentally observed post-barytocalcite phase. The double carbonate is significantly less stable than a mixture of the CaCO_3_ and BaCO_3_ end-members above 10 GPa. The experimental observation of the high-pressure phase up to 15 GPa and 300 ºC suggests that the decomposition into its single carbonate components is kinetically hindered.

## Introduction

Carbonate minerals play an important role in the geological carbon cycle. It is well-known that, in the process of subduction, carbon enters into the Earth’s mantle mainly in the form of carbonates, which are progressively subjected to increasing pressure and temperature as the subduction slab sinks^1^. Consequently, the determination of the influence of thermodynamic parameters such as pressure, temperature and composition on the stability and structural behavior of carbonates is key for geophysics.

The majority of experimental and computational investigations focused on simple magnesium (MgCO_3_ magnesite) and calcium (CaCO_3_ calcite and aragonite) carbonates and the double Mg-Ca dolomite carbonate because they are the most abundant on the Earth’s surface^2^ and their high-pressure (HP) high-temperature (HT) structures are thought to be the dominant host phases of carbon in the mantle^3–7^. However, in an environment of high compositional richness such as the Earth's mantle, the study of the effects of chemical substitution in the stability of the different carbonate structures is of primordial importance. Note, for instance, that a higher stability has been reported for double Ca-bearing carbonates (Ca–Mg dolomite, Ca–Fe ankerite, Ca–Ba alstonite) with respect to their corresponding single-cation minerals^8^.

Chemical stability is intimately related to the atomic arrangements within the structure. A paradigmatic example of chemical system with a complex energy landscape is BaCO_3_–CaCO_3_. Crystallization experiments in the system BaCO_3_–CaCO_3_–H_2_O constrained the solid solubility relations of the system. It has been reported that CaCO_3_ calcite could admit up to a 25% of Ba atoms in its structure, while BaCO_3_ witherite could admit up to 20% of Ca atoms^9^. This system is also characterized by the formation of, at least, five different polymorphs of the barium calcium BaCa(CO_3_)_2_ double carbonate^10–14^. These phases, namely barytocalcite (space group (SG): P2_1_/m)^10^, paralstonite (SG: P321)^11^, two variants of alstonite (SGs: P31m^12^ and P321^13^) and a synthetic monoclinic phase (SG: C2)^14^, have been identified at ambient conditions. According to recent theoretical DFT investigations, barytocalcite is the thermodynamically stable phase but the differences in enthalpies with the alstonite and paralstonite variants are very small (< 0.08 eV/formula unit), which suggests that either of these phases could be found in nature, as it occurs in fact^13^. Regarding the high-temperature structural behavior of barytocalcite, a phase transition to a disordered cation calcite form above 520 ºC has been reported^15^. The abundant polymorphism reveals the existence of numerous local energy minima in the BaCa(CO_3_)_2_ system and the need of exploring systematically its P–T phase diagram.

The complexity of the crystal chemistry of this Ba:Ca 1:1 double carbonate becomes particularly evident when examining the local environments around the cation atoms. In the CaCO_3_ calcite and aragonite structures, Ca atoms are coordinated by 6 and 9 oxygen atoms, respectively^16,17^. The Ba atoms in BaCO_3_ witherite are coordinated by nine oxygen atoms^17^. In the different BaCa(CO_3_)_2_ polymorphs the cation coordination varies significantly, between 6 and 8 for Ca atoms and 6 and 11 for Ba atoms. Thus, barytocalcite has the Ca and Ba atoms in 7- and 11-fold coordination by oxygen atoms, respectively^10^. The coordination of the cations in paralstonite and alstonite variants differ from barytocalcite, as Ba is coordinated by ten oxygen atoms and the Ca by eight oxygen atoms^11–13^. The synthetic phase^14^ and the HT disordered calcite phase^15^ present each Ba/Ca atom octahedrally coordinated. Note that Ca coordination environment in carbonates was previously reported to be related with chemical composition^18–21^ and to change upon compression^6,7,22,23^.

In order to give further insights into the crystal chemistry and the structural behavior of double BaCa(CO_3_)_2_ carbonates, we report in this work a joint high-pressure high-temperature experimental and theoretical investigation of the structural properties of barytocalcite. We characterized our sample by single-crystal and synchrotron powder X-ray diffraction (XRD) upon compression and found a novel dense polymorph above 5.5 GPa. The HP phase transition entails the increase of the coordination of certain Ca and Ba atoms which results in a more compact packing. We analyzed all the experimental results in the light of previously reported crystallographic data for BaCa(CO_3_)_2_ and of our DFT calculations.

## Results and discussion

### BaCa(CO_3_)_2_ barytocalcite structure at ambient conditions

**Figure 1:**
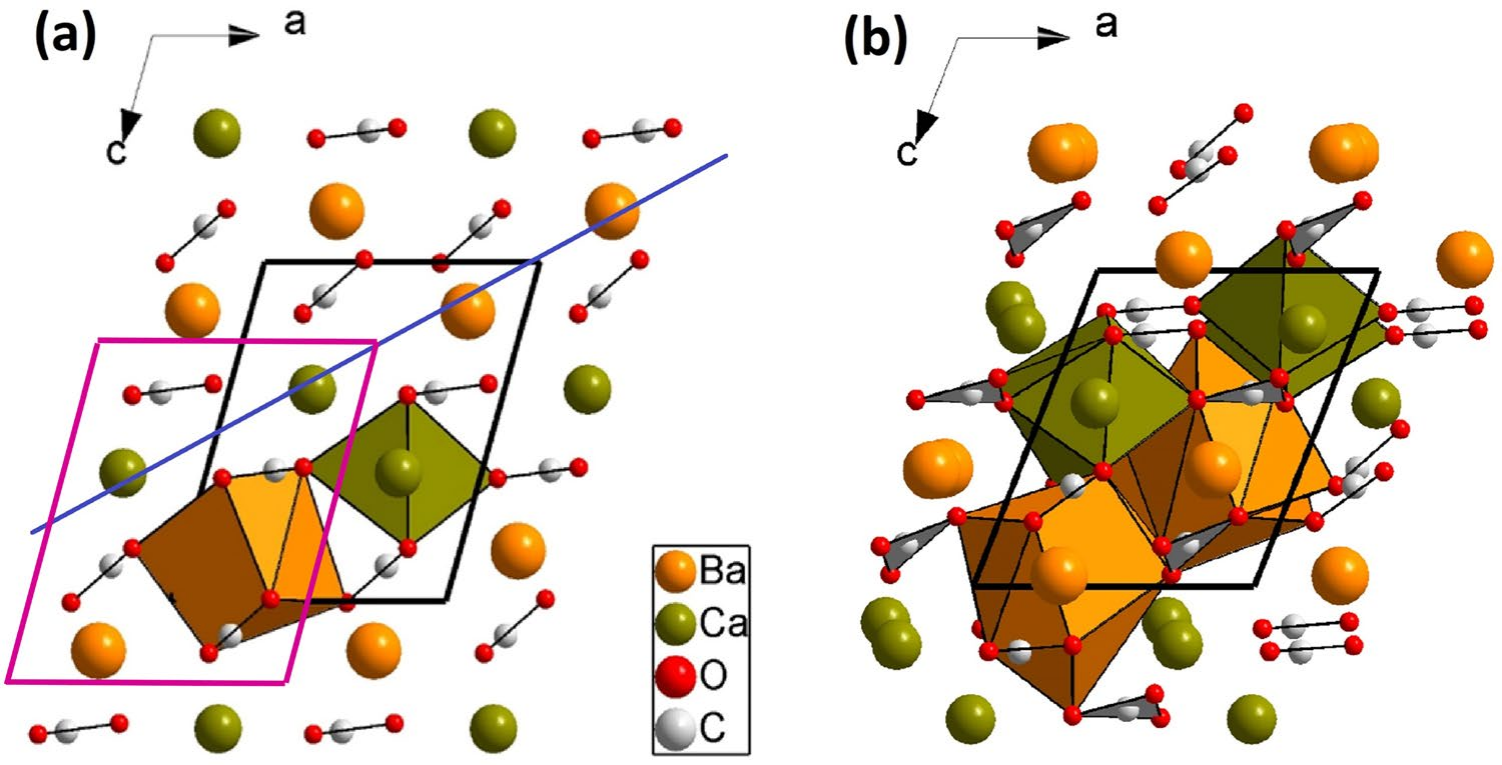
Structures of BaCa(CO_3_)_2_ *P*2_1_/*m* barytocalcite **(a)** and high-pressure *Pm* post-barytocalcite **(b)** projected along the *b* crystallographic unitcell axis. Note that the *b* axis of the high-pressure phase is twice that of the low-pressure barytocalcite. Orange, green, light gray, and red spheres represent Ba, Ca, C and O atoms, respectively. Cell edges are depicted as solid black lines. Magenta solid lines on the barytocalcite projection demarcate the location of post-barytocalcite unit cell contents, for the sake of comparison between structures. The diagonal blue line indicates a hexagonal layer of metallic atoms.

Single-crystal XRD data confirms that our initial sample is BaCa(CO_3_)_2_ barytocalcite, as reported by Dickens and Bowen^10^. Our analysis shows that the data at 1 atm and 20 °C can be refined in the centrosymmetric *P*2_1_/*m* space-group with lattice parameters: *a* = 6.5503(2) Å, *b* = 5.2435(2) Å, *c* = 8.1091(3) Å, and *β* = 106.019(4)° (V = 267.699(17) Å^3^), which are in good agreement with previously reported cell dimensions^10^. The experimental atomic coordinates collected in Table 1 are also similar to those previously determined, describing the barytocalcite structure depicted in Fig. 1a. The good agreement with our theoretically calculated values is also shown in Table 1. The topology of the atomic arrangement in barytocalcite has been discussed elsewhere, but, for the sake of comparison, it will be briefly described here.

**Table 1:** Experimental (single-crystal, *sc*) and calculated (DFT) lattice parameters and atomic coordinates of BaCa(CO_3_)_2_ barytocalcite structure at ambient conditions.

		*sc*-BaCa(CO_3_)_2_ *a* = 6.5503(2) Å *b* = 5.2434(2) Å *c* = 8.1091(3) Å *β* = 106.019(4)º V = 267.699(17) Å^3^			*DFT*-BaCa(CO_3_)_2_ *a* = 6.5528 Å *b* = 5.2611 Å *c* = 8.1095 Å *β* = 106.391º V = 268.213 Å^3^		
Atom	Wyckoff position	x	y	z	X	y	z
Ba	2e	0.21171(4)	0.75	0.85259(4)	0.21022	0.75	0.85054
Ca	2e	0.69843(15)	0.25	0.62303(12)	0.69546	0.25	0.62374
C1	2e	0.7482(7)	0.75	0.8970(6)	0.74787	0.75	0.89950
C2	2e	0.7533(7)	0.75	0.3855(6)	0.74608	0.75	0.38673
O1	2e	0.6400(5)	0.75	0.0050(5)	0.64009	0.75	0.01026
O2	4f	0.8086(4)	0.5391(4)	0.8452(3)	0.80824	0.53724	0.84645
O3	4f	0.6528(4)	0.9608(4)	0.3937(3)	0.65180	0.96156	0.39375
O4	2e	0.9362(5)	0.75	0.3616(5)	0.94013	0.75	0.36695

The structure is described with the monoclinic *P*2_1_/*m* space group.

Barytocalcite presents slightly corrugated pseudo-hexagonal layers of cations parallel to the *b* axis (see Fig. 1a), each layer being formed by atoms of Ca and Ba in the same proportion. If the difference between Ca and Ba is ignored, the cation layers roughly repeat every third layer as in calcite. The orientations of the CO_3_ groups in both calcite and barytocalcite, however, differ significantly and cause the huge differences between Ba and Ca coordinations. Ba atoms occupy a position between 6 carbonate groups, with a Ba coordination sphere formed by 11 oxygen atoms (5 [CO_3_] edges + 1 [CO_3_] corner), and the Ca atoms are also surrounded by 6 carbonate groups, but having 7 oxygen neighbors (1[CO_3_] edge + 5 [CO_3_] corners).

### Compressibility of the BaCa(CO_3_)_2_ barytocalcite structure

**Figure 2:**
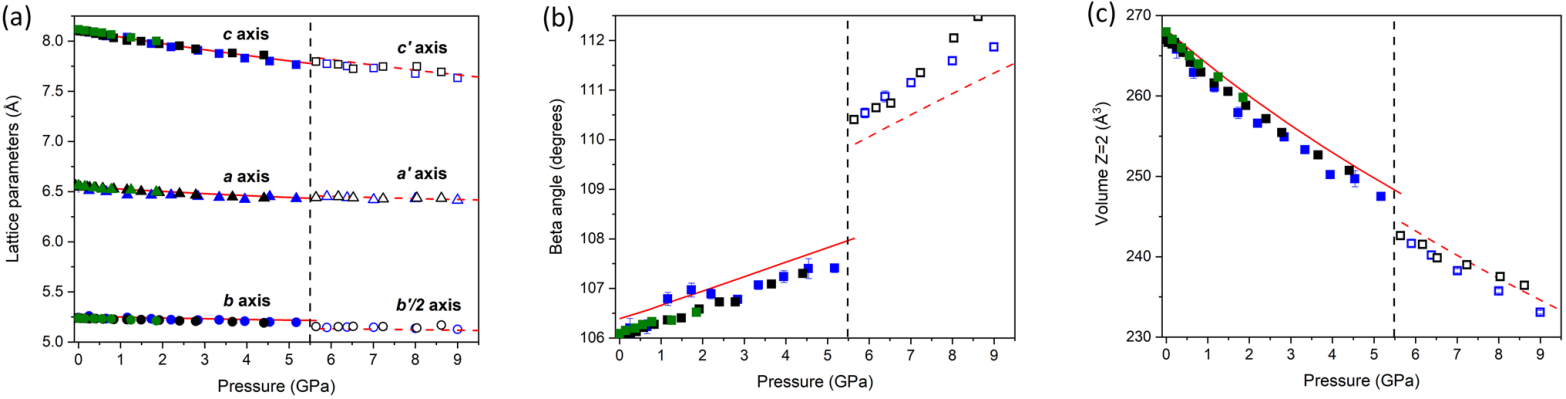
(**a**) Pressure dependence of the lattice parameters *a* (triangles) and *c* (squares) of low-pressure *P*2_1_/*m* barytocalcite (solid symbols) and high-pressure *Pm* post-barytocalcite (empty symbols) BaCa(CO_3_)_2_ phases. Circles represent the *b* barytocalcite axis but half of the *b*’ axis of post-baritocalcite. Blue symbols correspond to single-crystal data using a methanol:ethanol mixture as pressure transmitting medium, while black and green symbols correspond to two different powder runs using silicone oil as pressure medium. Solid and dashed red lines are fits to theoretical DFT data of the low- and high-pressure phases, respectively. The vertical dashed line indicates the transition pressure. (**b**) Evolution of the monoclinic *β* angle according to experiments and calculations. (**c**) Pressure dependence of the volume per 2 formula units of the two BaCa(CO_3_)_2_ phases.

**Figure 3:**
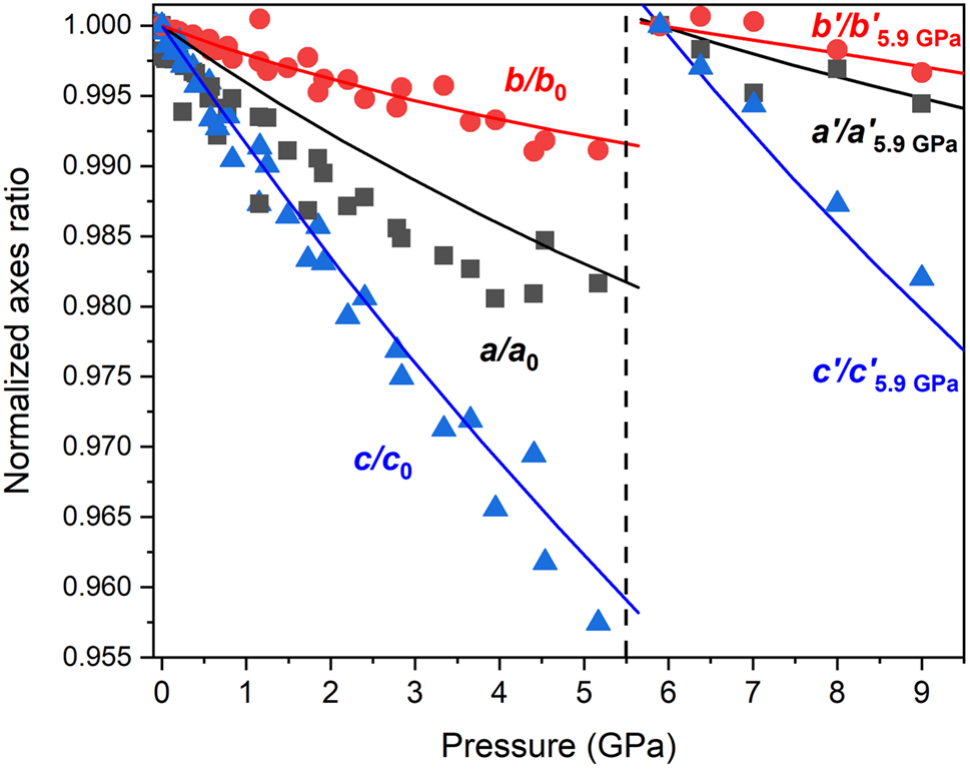
Normalized lattice parameters of BaCa(CO_3_)_2_ barytocalcite and HP post-barytocalcite phases as a function of pressure, which provide the information on axial compressibilities.

Both the in situ single-crystal and powder XRD patterns of BaCa(CO_3_)_2_ barytocalcite at different pressures could be indexed with the monoclinic *P*2_1_/*m* barytocalcite structure stable at ambient conditions up to 5.2 GPa (Tables 1S and Table 2S of Supplementary Material). HP powder synchrotron XRD data present intensities that do not correspond to perfect randomly oriented powder, so only peak positions and not relative intensities could be used to the structural analysis. In other words, from powder diffraction data, we could only accurately infer the lattice parameters of the mineral upon compression. HP single-crystal XRD measurements allow us to fully characterize the progressive transformations of the barytocalcite structure with increasing pressure. Table 1S of Supplementary Material collects the details and parameters of the single-crystal X-ray diffraction refinements to illustrate their quality. From the diffraction patterns collected at different pressures and our DFT calculations (Table 3S) we extracted the pressure evolution of the lattice parameters of this phase. The obtained evolution for the unit-cell parameters and the cell volume are plotted in Fig. 2. The axial compressibilities, defined as *κ* =  *− 1/x(∂x/∂P)* (where *x* = *a, b, c*), estimated from our experimental (theoretical) data are *κ*_*a0*_ = 3.8(2) × 10^−3^ GPa^−1^ (3.29(9) × 10^−3^ GPa^−1^), *κ*_*b0*_ = 1.80(11) × 10^−3^ GPa^−1^ (1.50(7) × 10^−3^ GPa^−1^) and *κ*_*c0*_ = 8.0(2) × 10^−3^ GPa^−1^ (7.41(15) × 10^−3^ GPa^−1^), which evidence the strong anisotropy in this compound. Figure 3 clearly shows that the least compressible axis is the *b*-axis. This response to external pressure arises from the fact that the relatively incompressible [CO_3_] carbonate units are arranged parallel to the *b* axis, whereas the compressibility of the *a* and *c* axes is directly attributable to the compression of [CaO_7_] and [BaO_11_] polyhedra (see Fig. 1a).

A third-order Birch−Murnaghan (BM) EOS was fitted to all our pressure−volume data sets, including HP single-crystal and synchrotron powder XRD data (Fig. 2), yielding a zero-pressure unit-cell volume (V_0_), a bulk modulus (B_0_) and its first pressure derivative (B′_0_) of V_0_ = 267.5(2) Å^3^, B_0_ = 57(3) GPa, and B′_0_ = 5(2), respectively. These values are in good agreement with those obtained from our calculations: V_0_ = 268.21(1) Å^3^, B_0_ = 59.98(8) GPa, and B′_0_ = 4.43(2). The compressibility of barytocalcite lies in between those of the two end-member carbonates: 67(2) GPa for CaCO_3_ calcite (B′_0_ = 4)^24^, 66.5(7) GPa (B′_0_ = 5.0(1)) for CaCO_3_ aragonite^25^ and 48(1) GPa for BaCO_3_ witherite^26^, and it is comparable to the 62.7(6) GPa of SrCO_3_ strontianite^26,27^. Barytocalcite’s bulk modulus is slightly smaller than that of other BaCa(CO_3_)_2_ polymorph, alstonite, which is reported to have an experimental value of B_0_ = 60(3) GPa^13^. Theoretical compressibility results on alstonite using the same methodology (B_0_ = 62.8(2) GPa) confirm this smaller B_0_ value for barytocalcite. In other words, our data evidences that BaCa(CO_3_)_2_ barytocalcite is the most compressible of all the divalent metal carbonates and silicate-carbonates except witherite.

**Figure 4:**
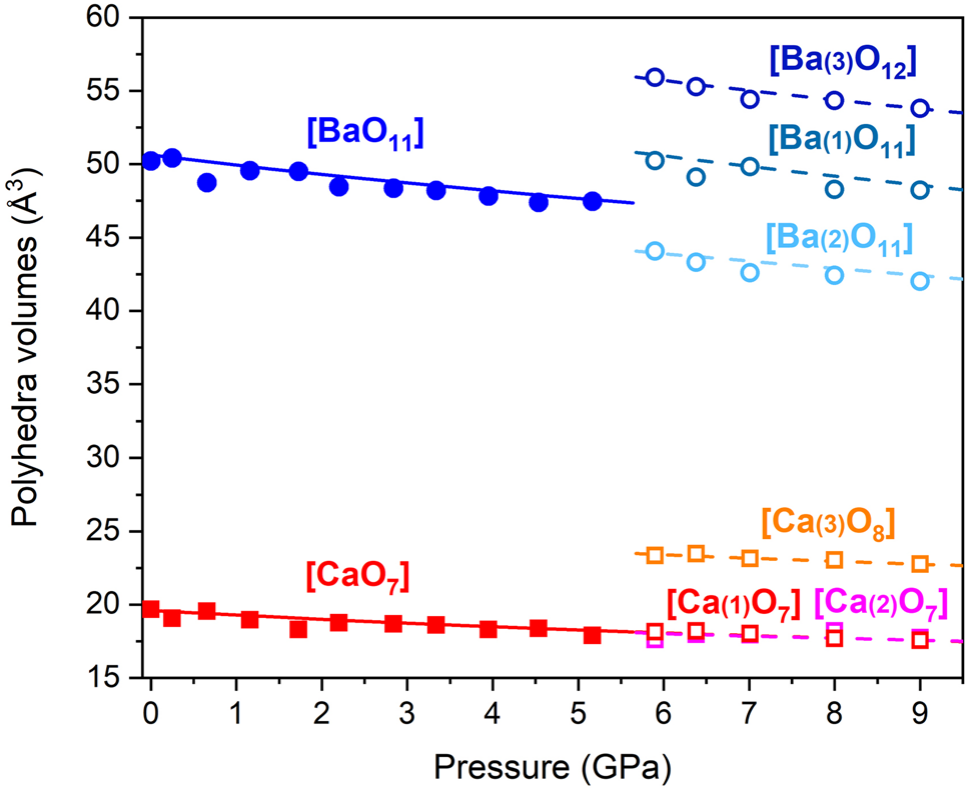
Representation of the evolution under ambient-temperature compression of the different cation-centered polyhedral unit volumes of BaCa(CO_3_)_2_ barytocalcite and HP post-barytocalcite phases according to single-crystal experiments and DFT calculations. At the transition (5.5 GPa), atoms rearrange changing their coordination geometries. ¼ of the Ba atoms (Ba(3)) and ½ of the Ca atoms (Ca(3)) increase their coordination number from 11 and 7 to 12 and 8, respectively.

From single-crystal XRD refinements and DFT calculations we determined the evolution of the atomic coordinates and the continuous modification of the structural arrangement of barytocalcite with increasing pressure. There is only one Ca site and one Ba site in barytocalcite with the aforementioned 7- and 11-fold coordinations, respectively. The analysis of cation-centered polyhedra in terms of the Voronoi-Dirichlet formalism^28^ confirms that the number of first neighbor atoms around Ca and Ba cations did not change while in the barytocalcite phase. The smooth decrease of the [CaO_7_] and [BaO_11_] polyhedral volumes upon compression is plotted in Fig. 4, with an experimental (theoretical) volume reduction of 9% (6.9%) and 5.5% (6.0%), respectively, between ambient pressure and 5.2 GPa.

### Dense BaCa(CO_3_)_2_ post-barytocalcite polymorph

A phase transition occurs between 5.2 and 5.7 GPa. According to single-crystal XRD measurements, the initial BaCa(CO_3_)_2_ barytocalcite phase is observed at 5.2 GPa and the new dense carbonate polymorph was observed at 5.9 GPa, the following pressure data-point. In powder XRD measurements the transition occurs between 4.4 and 5.7 GPa. Taking the results of both experiments into account, the transition pressure is estimated to be 5.5(3) GPa. Single-crystal XRD measurements allow us to fully characterize the nature of the high-pressure phase of the Ba-Ca double carbonate. Above 5.7 GPa, the crystal structure can no longer be described by the barytocalcite *P*2_1_/*m* space group, but by the lower symmetry *Pm* space group. The refinement of the structure included all the atomic coordinates and the isotropic displacement parameters. Data pertinent to the intensity data collection are summarized in Supplementary Table 4S. The lattice parameters of the high-pressure phase at 5.9 GPa are *a’* = 6.4510(10) Å, *b’* = 10.2907(7) Å, *c’* = 7.775(3) Å, and *β’* = 110.54(10)º (V’ = 483.3(8) Å^3^). Post-barytocalcite is the result of atomic rearrangement in the initial BaCa(CO_3_)_2_ barytocalcite structure to deal with repulsive interactions at high density, the phase transition occurring to minimize the overall enthalpy of the system. Although the transition comes with a volume collapse of ∼1.4%, the symmetry of the initial and final structures is related by a group-subgroup relationship and the cooperative atomic displacements can be easily tracked. The final positional parameters of *Pm* post-barytocalcite at 9 GPa are given in Table 2 together with those of initial barytocalcite described with the same *Pm* space group. For this, the *P*2_1_/*m* space group of the aristotype barytocalcite was firstly transformed into the *translationengleich Pm* subgroup with the same initial lattice parameters *a, b, c* and *β*. Subsequently, the *b* axis is doubled through a *klassengleiche IIc* transformation to final lattice parameters *a, 2b, c* and *β* (similar in dimensions to the *a’, b’, c’* and *β’* parameters of the HP phase). To compare the atomic coordinates of this *Pm*-described barytocalcite phase with those of the HP phase, an (0.5, 0, −0.25) origin shift is applied. As can be seen in Table 2, the symmetry reduction causes that the Wyckoff position 2e occupied with Ba, Ca, C and O atoms in the *P*2_1_/*m* barytocalcite structure, once the cell is doubled, split into three symmetrically independent positions: 2c, 1a and 1b. The O atoms initially located in 4f positions are placed in four 2c Wyckoff positions. Taking this into account, the analysis of the atomic arrangements of both the low- and the high-pressure phases is simpler and the structural differences can be easily found.

**Table 2:** Experimental atomic coordinates (x, y, z) of the initial barytocalcite structure at ambient conditions described with the *Pm* space group (once the symmetry reduction, cell doubling and origin shift mentioned in the main text are applied; *a* = 6.5503(2) Å, *b* = 10.4868(4) Å, *c* = 8.1091(3) Å, *β* = 106.019(4)º) to be compared with those of the high-pressure *Pm* experimental post-barytocalcite phase at 9 GPa (*a’* = 6.4510(10) Å, *b’* = 10.2907(7) Å, *c’* = 7.775(3) Å, and *β’* = 110.54(10)º) denoted as (x_HP-Exp_, y_HP-Exp_, z_HP-Exp_) and the theoretically-calculated post-barytocalcite phase at 9.47 GPa (*a’* = 6.4168 Å, *b’* = 10.232 Å, *c’* = 7.6427 Å, and *β’* = 111.53º) denoted as (x_HP-Th_, y_HP-Th_, z_HP-Th_).

Atom	Site	x	x_HP-Exp_	x_HP-Th_	y	y_HP-Exp_	y_HP-Th_	Z	z_HP-Exp_	z_HP-Th_
Ba1	2c	0.28829	0.2909(5)	0.288443	0.25	0.2347(10)	0.233927	0.89741	0.9655(2)	0.965687
Ba2	1b	0.71171	0.7328(7)	0.735703	0.5	0.5	0.5	0.60259	0.6237(3)	0.627349
Ba3	1a	0.71171	0.6894(7)	0.686545	0	0	0	0.60259	0.6309(3)	0.632645
Ca1	1a	0.8016	0.810(3)	0.807415	0	0	0	0.1270	0.1843(13)	0.184965
Ca2	1b	0.8016	0.739(3)	0.740071	0.5	0.5	0.5	0.1270	0.1122(13)	0.115678
Ca3	2c	0.1984	0.178(2)	0.175598	0.25	0.2721(4)	0.27243	0.3730	0.4201(10)	0.420234
O1	2c	0.6914	0.688(8)	0.68771	0.1445	0.1500(14)	0.147771	0.9048	0.960(3)	0.958241
O2	2c	0.6914	0.614(7)	0.614176	0.6445	0.6440(13)	0.643646	0.9048	0.872(3)	0.877279
O3	2c	0.1528	0.089(7)	0.083389	0.6054	0.6079(12)	0.608141	0.1437	0.133(3)	0.133957
O4	1b	0.4362	0.387(10)	0.372104	0.5	0.5	0.5	0.1116	0.111(5)	0.104076
O5	1b	0.14	0.298(11)	0.295325	0.5	0.5	0.5	0.755	0.501(5)	0.500181
O6	2c	0.3086	0.149(6)	0.149473	0.3945	0.3931(12)	0.391392	0.5952	0.680(3)	0.680000
O7	2c	0.8472	0.851(7)	0.842145	0.8554	0.8504(13)	0.852083	0.3563	0.416(3)	0.415168
O8	2c	0.8472	0.796(7)	0.795646	0.3554	0.3539(13)	0.356664	0.3563	0.340(3)	0.344629
O9	1a	0.14	0.123(10)	0.131765	0	0	0	0.755	0.794(4)	0.801039
O10	2c	0.3086	0.300(8)	0.298982	0.8945	0.8928(13)	0.891158	0.5952	0.635(3)	0.635888
O11	1a	0.4362	0.436(12)	0.438769	0	0	0	0.1116	0.186(5)	0.190195
O12	2c	0.1528	0.146(7)	0.142244	0.1054	0.1072(14)	0.107459	0.1437	0.205(3)	0.207164
O13	2c	0.86	0.844(9)	0.84634	0.25	0.2434(11)	0.244763	0.745	0.778(4)	0.777262
O14	2c	0.5638	0.53513)	0.530738	0.25	0.2516(12)	0.250087	0.3884	0.408(6)	0.411196
C1	2c	0.7518	0.701(13)	0.720607	0.25	0.2502(16)	0.249582	0.853	0.866(6)	0.872776
C2	1b	0.2533	0.196(16)	0.185273	0.5	0.5	0.5	0.1355	0.126(7)	0.126665
C3	1b	0.2482	0.201(14)	0.203575	0.5	0.5	0.5	0.647	0.626(6)	0.625753
C4	2c	0.7467	0.719(14)	0.717636	0.25	0.2506(16)	0.250519	0.3645	0.388(6)	0.389442
C5	1a	0.2482	0.214(16)	0.238657	0	0	0	0.647	0.681(6)	0.687927
C6	1a	0.2533	0.256(16)	0.246648	0	0	0	0.1355	0.206(7)	0.204882

Figure 1b shows the projection of the high-pressure phase along the *b* crystallographic axis, which illustrates three different types of cation-centered oxygen polyhedra for Ba and Ca (corresponding to atoms located at 2c, 1a and 1b sites). At the transition, one fourth of the Ba atoms increase their coordination number to 12, and the rest remain with 11 O neighbors. Regarding the Ca atoms, half of them increase their coordination number from 7 to 8. The increase in the coordination numbers of cations across the pressure-induced phase transition was expected according the pressure-coordination rule^29^. The coordination sphere around the C atoms has not changed, remaining in trigonal planar configuration. The HP phase presents, therefore, an unusual variety of polyhedral geometries and volumes within the same structure and it stands as a possible host phase for other divalent cations in Earth’s mantle without inducing significant elastic strains. The appearance of such variety of coordination polyhedra is a consequence of the displacement, tilting and rotation of the [CO_3_] carbonate groups. In the initial barytocalcite structure, the [CO_3_] groups have 4 different orientations, all the carbonate units lying along the *b* axis and having the same orientation (see Fig. 1Sa of Supplementary Material). In the HP phase, however, the disappearance of the 2_1_ symmetry screw axes and the doubling of the unit cell along the *b* direction allow the carbonate groups to locate at different positions and to adopt a range of different orientations (see Fig. 1Sb). At the transition, most of the atoms displace by less than 0.3 Å, but one carbonate group [C(3)O_3_] rotates approximately 60º. These displacements entail the aforementioned coordination change in ¼ and ½ of the Ba and Ca atoms, respectively.

### Compressibility of the BaCa(CO_3_)_2_ post-barytocalcite structure

From the X-ray diffraction data and DFT calculations, we obtained the evolution with pressure of the unit-cell lattice parameters and volume of the HP post-barytocalcite phase (Tables S4–S6 of Supplementary Material). The theoretical data were analyzed using a third-order BM EOS. In the analysis of the experimental data, due to the small number of available P–V data points, we fixed the value of the theoretical bulk modulus first pressure derivative (B_0_′ = 4.53(3)). The fit to the experimental single-crystal (theoretical) data yielded a zero-pressure volume V_0_ = 528.8(8) Å^3^ (V_0_ = 534.44(12) Å^3^) and a bulk modulus B_0_ = 53.6(10) GPa (B_0_ = 51.4(2) GPa) for the high-pressure phase. Therefore, the HP polymorph has a slightly larger compressibility consequence of the new atomic distribution. To give further insight into this behavior, we first analyzed the evolution of the experimentally obtained and DFT-calculated lattice parameters with compression and, subsequently, the evolution of the polyhedral unit volumes. The experimental (theoretical) axial compressibilities of the HP phase *κ*_*a*_ = 1.5(6) × 10^−3^ GPa^−1^ (1.59(4) × 10^−3^ GPa^−1^), *κ*_*b*_ = 1.2(3) × 10^−3^ GPa^−1^ (0.956(5) × 10^−3^ GPa^−1^) and *κ*_*c*_ = 5.9(2) × 10^−3^ GPa^−1^ (6.24(16) × 10^−3^ GPa^−1^) are slightly smaller than those of the low-pressure phase, but the beta angle increases at a higher rate upon compression (see Figs. 2 and 3). Taking into account the good agreement found between experimental and theoretical data in the lattice parameters and atomic positions of both the LP and HP phases (see Tables 1 and 2) and in the unit-cell compressibility data, we used the less scattered data from our ab initio total-energy simulations to study the variation in polyhedral compressibility with pressure. The compression of the unit cell is dominated by the cation-centered polyhedral units. In the LP barytocalcite phase (see Fig. 4), the seven-fold [CaO_7_] capped-octahedra are the most compressible units with a bulk modulus, 60.8(4) GPa, similar to that of the unit cell, and [BaO_11_] polyhedra are slightly less compressible with a bulk modulus of 70.1(7) GPa. The compressibility of the HP phase is governed, however, by the compressional behavior of [BaO_X_] polyhedra with bulk moduli between 42 and 53 GPa, the [CaO_X_] polyhedra having bulk moduli between 70 and 85 GPa. Note that besides the diversity of cation environments defined by the number of oxygen neighbors and their topology, this polymorph also presents a range of polyhedral compressibilities.

The phase transition is fully reversible. It occurs somewhere between 5.1 and 3.6 GPa in the decompression process, showing therefore an appreciable hysteresis using silicone oil as pressure transmitting medium. The recovered sample has the initial barytocalcite structure and unit cell dimensions.

### High-pressure high-temperature behavior of the barytocalcite structure

In order to estimate the thermal expansion of BaCa(CO_3_)_2_ barytocalcite at high pressure and evaluate the role of temperature in the HP structural phase transformation, we performed an externally resistive-heating DAC experiment (up to 310 ºC) using synchrotron XRD to characterize in situ the sample. In our experimental run, the sample was initially compressed to 1.8 GPa, subsequently heated to 310 ºC, then isothermally compressed up to 15 GPa and, finally, the pressure and the temperature were quenched to ambient conditions. Note that the sample is maintained well below the decarbonation onset temperature of 600 ºC for this compound^30^.

**Figure 5:**
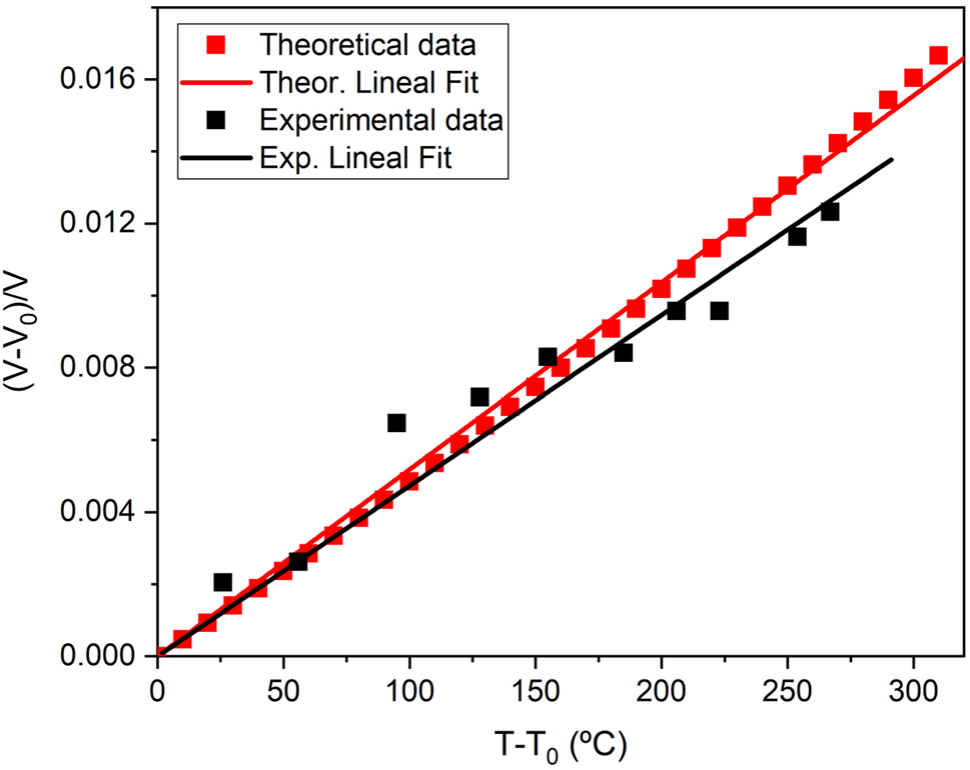
Evolution of the experimental (black) and theoretical (red) relative volume change of BaCa(CO_3_)_2_ barytocalcite at ~ 3 GPa as a function of temperature. The slope of the linear fit (solid line) provides an estimation of the average thermal expansion for barytocalcite between 20 and 300 ºC.

During the heating process, the pressure in the sample chamber increases slightly with increasing temperature, from 1.8 GPa at ambient temperature to 3.1 GPa at the highest temperature. Powder XRD patterns suggest that no structural transformations take place. Since our temperature-volume data do not correspond to an isobar, we estimated the thermal expansion at ~ 3 GPa using the ambient-temperature EOS to estimate a reference unit cell volume at each pressure that was subtracted from the unit cell volume at high temperature and subsequently normalized to this latter volume. Therefore, the slope of a plot of (V-V_ref_)/V versus (T − T_ref_) is a measure of the volumetric thermal expansion (see Fig. 5). A linear fit to our experimental data yields a thermal expansion value of α_Exp, 3 GPa_ = 4.7(2) × 10^−5^ ºC^−1^, in good agreement with the DFT calculated value at the same pressure (α_Th, 3 GPa_ = 5.18(3) × 10^−5^ ºC^−1^). These results for thermal expansion of the Ba-Ca double carbonate at 3 GPa can be compared to that of the corresponding simple end-member carbonates at ambient pressure found in literature. That is, 4.10(7) × 10^−5^ ºC^−1^^31^, 4.9(2) × 10^−5^ ºC^−1^^25^ and 5.7(2) × 10^−5^ ºC^−1^^32^ for calcite, aragonite and witherite, respectively.

Upon isothermal (310 ºC) compression, the barytocalcite structure undergoes the transition to the HP *Pm* post-barytocalcite phase between 5.2 and 5.7 GPa. This transition pressure is similar to that at ambient temperature. No accurate information on the Clapeyron slope of the phase boundary could be obtained given the fact that our two P–T data points have large uncertainties (transition pressures of 5.5(3) and 5.5(3) GPa for temperatures of 20(1) ºC and 310(4) ºC, respectively), but the transition pressure seems not to be significantly affected by high temperatures in the studied temperature range. No additional evident phase transition was observed during the pressure upstroke, the initial barytocalcite phase being recovered after pressure and temperature quenching at ambient conditions.

### Relative stability of the different BaCa(CO_3_)_2_ phases

**Figure 6:**
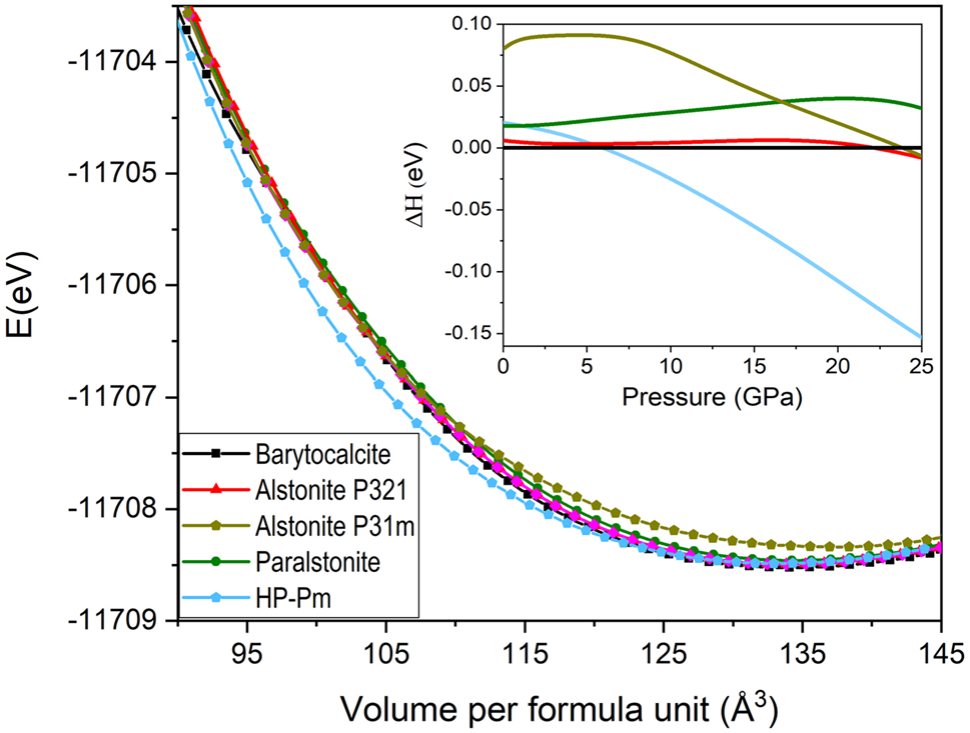
Cohesive energy as a function of the volume per BaCa(CO_3_)_2_ formula unit for the *P*2_1_/*m* barytocalcite and HP *Pm* post-barytocalcite phases, but also *P*321 alstonite^13^, *P*31*m* alstonite^12^ and *P*321 paralstonite^11^ phases. Inset: Enthalpy difference as a function of pressure, showing the stabilities with respect to *P*2_1_/*m* BaCa(CO_3_)_2_ barytocalcite.

To get further insight into the relative thermodynamic stability of the different BaCa(CO_3_)_2_ polymorphs and their pressure-induced transformations, we have performed ab initio total-energy calculations of the *P*2_1_/*m* barytocalcite, the high-pressure *Pm* post-barytocalcite, *P*321 alstonite^13^, *P*31*m* alstonite^12^, P321 paralstonite^11^ mineral phases and the synthetic *C*2 phase^14^. Figure 6 shows the calculated curves for energy as a function of volume, and the enthalpies calculated for each phase referring to the enthalpy of barytocalcite are shown in the inset. According to our PBEsol calculations, barytocalcite is the most stable phase amongst those considered at ambient conditions. Note, however, that the enthalpies of the barytocalcite and *P*321 alstonite^13^ phases are very similar below 20 GPa (within 0.007 eV per formula unit of one another), the barytocalcite enthalpy curve lying below that of alstonite. For *P*321 paralstonite^11^, the high-pressure *Pm* post-barytocalcite and *P*31*m* alstonite^12^, we obtain enthalpies per formula unit 0.018 eV, 0.02 eV and 0.08 eV higher than that of barytocalcite, respectively. Such small energy differences between polymorphs are consistent with the fact that the four of them are found in nature as minerals. The synthetic *C*2 phase is highly unstable at all pressures relative to any of the other phases (~ 0.29 eV higher that barytocalcite per formula unit at ambient conditions, the enthalpy difference increasing with pressure). From the enthalpy-pressure curves plotted in the inset of Fig. 6, we can infer a pressure-induced phase transition of barytocalcite to our HP post-barytocalcite phase at 5.9 GPa, in relative good agreement with the experimental data where the phase transition occurred between 5.2 and 5.7 GPa. Therefore, the HP phase becomes the thermodynamically stable phase above that pressure, the enthalpy difference with respect to other phases continuously increasing upon further compression.

**Figure 7:**
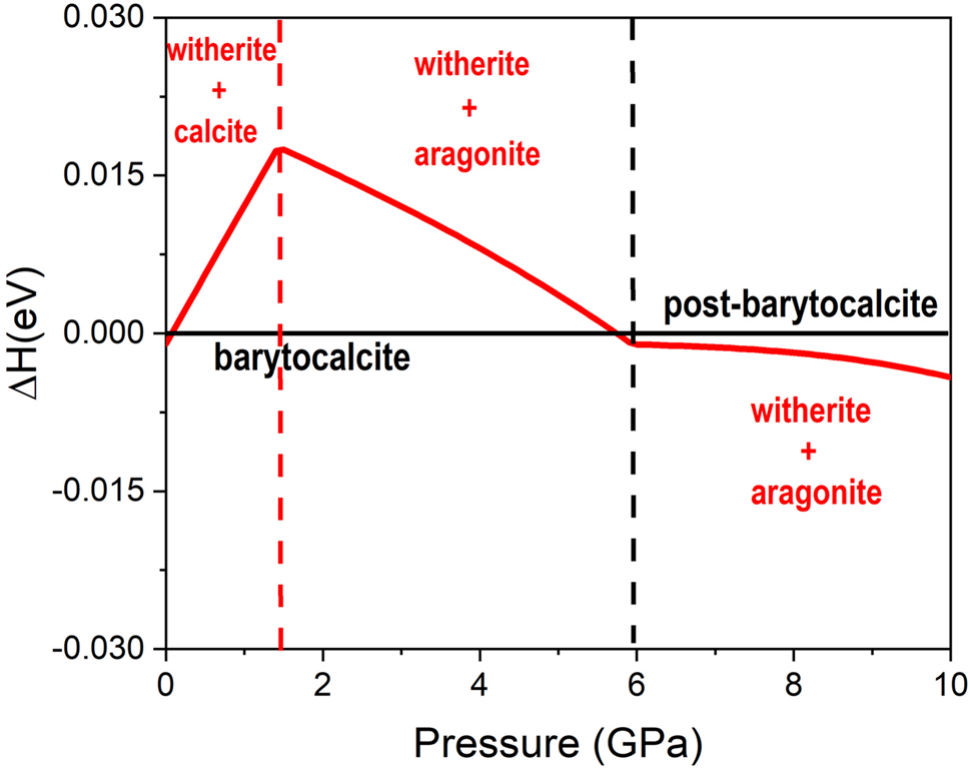
Calculated enthalpy difference between the mixture of single carbonate phases (BaCO_3_ + CaCO_3_) and the most stable phase for the double BaCa(CO_3_)_2_ as a function of pressure. Relevant polymorphs at each pressure were considered.

We have also studied the stability of the BaCa(CO_3_)_2_ double carbonate with respect to that of the corresponding end member simple carbonates, CaCO_3_ + BaCO_3_. For this purpose, we carried out additional calculations on the *R*3*c* CaCO_3_ calcite, *Pmcn* CaCO_3_ aragonite, *Pmcn* BaCO_3_ witherite and HP *P*-31*c* BaCO_3_-II^33^ phases. Figure 7 shows the sum of the enthalpies of the thermodynamically stable simple carbonate polymorphs relative to the enthalpy of thermodynamically stable BaCa(CO_3_)_2_ phase at each pressure. In the 0–1.4 GPa pressure range, barytocalcite is more stable that a calcite + witherite mixture and the enthalpy difference increases with pressure. At 1.4 GPa, according to our calculations, calcite transforms into aragonite and, above this pressure, the sum of enthalpies of aragonite + witherite gets closer to that of barytocalcite. These results are in agreement with a previous study which reported that BaCa(CO_3_)_2_ alstonite is more stable than their respective end members^8,34^. From Fig. 7, we also see that the aragonite + witherite mixture is thermodynamically more stable above 5.8 GPa. At 5.9 GPa, the barytocalcite to HP post-barytocalcite transformation occurs in BaCa(CO_3_)_2_, and, from this pressure to 10 GPa, the slope of enthalpy difference changes to approximately horizontal with a very small and constant energy difference of 3 meV per formula unit. Above 10 GPa, the mixture of simple carbonates becomes increasingly more stable upon further compression.

## Conclusions

In situ HP and HP-HT XRD experimental investigations on powder and single-crystals from a natural BaCa(CO_3_)_2_ barytocalcite mineral sample show that the initial monoclinic *P*2_1_/*m* structure is stable up to approximately 5.5(3) GPa in the 20–310 ºC temperature range. Further compression at room temperature reveals a phase transition towards a *Pm* post-barytocalcite structure, which involves the increase in coordination number of several Ba and Ca atoms and differs in polyhedral connectivity from the original barytocalcite structure. Structural differences derive from the decrease in lattice symmetry, which allows symmetry-unconstrained displacements and rotations. Thus, most of the [CO_3_] carbonate groups appear tilted with small atomic displacements (< 0.3 Å) with respect to the initial barytocalcite phase, but one eighth of them rotate approximately 60º, which causes major changes in one fourth and one half of the Ba and Ca polyhedra, respectively. This increases the coordination number of the 11-fold Ba and sevenfold Ca atoms to 12 and 8 O atoms, respectively. This phase transformation can be tracked by means of a group-subgroup symmetry relationship that yields information on the plausible transition mechanism and might help to better understand the crystallography of yet unknown carbonate polymorphs.

The observations of the experimental study are consistent with DFT calculations that confirm the reported structural transformation, the experimental barytocalcite thermal expansion, the barytocalcite and post-barytocalcite compressibility and anisotropy, and give an overall picture of the energy landscape within the BaO–CaO–CO_2_ system. In this sense, our ab initio simulations indicate that the BaCa(CO_3_)_2_ double carbonate is thermodynamically more stable than the mixture of simple end member CaCO_3_ and BaCO_3_ carbonates below 5.8 GPa. Between 5.8 and 10 GPa, both options are energetically similar (enthalpies per formula unit within less 3 meV) and, above this pressure, the double carbonate decomposition into its simple carbonate components is clearly favored. Further experiments and calculations are needed to determine the thermodynamic crossovers of this and other carbonate systems and constrain the pressure–temperature conditions at which simple and double carbonates could exist at inner Earth conditions.

## Methods

### Experimental details

Naturally occurring barytocalcite crystals from Alston Moor, in Cumbria (U.K.), were kindly provided by the Yale Peabody Museum (Specimen YPM MIN 056782). A few crystals were optically selected under the microscope. Some were chosen to undertake single-crystal X-ray diffraction experiments whereas others were crushed to obtain a fine white powder. Qualitative chemical analyses were done on a Philips XL30 scanning electron microscope using energy-dispersive X-ray spectroscopy. According to them, the chemical composition of our barytocalcite sample was Ba_1.02(3)_Ca_0.98(3)_(CO_3_)_2_, nominally BaCa(CO_3_)_2_. We solved the barytocalcite structure at ambient conditions from angle-dispersive single-crystal XRD data collected on a Rigaku SuperNOVA diffractometer equipped with an EOS CCD detector and Mo radiation micro-source (λ = 0.71073 Å). The measurement was processed with the CrysAlisPro software version 1.171.41.117^35^. This software was used to collect, index, scale and apply numerical absorption correction based on gaussian integration over a multifaceted crystal model and empirical absorption correction using spherical harmonics, implemented in SCALE3 ABSPACK scaling algorithm into CrysAlisPro. The structure was solved applying the novel dual-space algorithm implemented in SHELXT program^36^. Fourier recycling and least-squares refinement were used for the model completion with SHELXL-2018^37^. The obtained barytocalcite structure can be described with a monoclinic *P*2_1_/*m* space-group and it is in good agreement with that reported by Dickens and Bowen^10^. This structure was briefly described in the text, since it is the initial phase of a series of pressure-induced modifications.

High-pressure XRD data were acquired in two ways. HP powder XRD measurements were carried out at ALBA Synchrotron (Barcelona, Spain) on the BL04—MSPD beamline^38^ using a monochromatic beam of wavelength 0.4246 Å focused to a spot size of 20 × 20 μm^2^ at half of its maximum intensity. A SX165 Rayonix Mar CCD detector was used to record the data. For HP experiments we used a membrane-driven diamond anvil cell (DAC) with diamond culets of 450 microns (RT) and 300 microns (HT), a technique that allows compressing materials and characterizing them in situ while compressed^39,40^. In the high-pressure room-temperature run, the sample was loaded in a 125 to 150 μm diameter and 40 μm-thick stainless steel chamber together with silicone oil, used as quasi-hydrostatic pressure-transmitting medium^41^, and elemental copper, used as internal pressure gauge^42^. In the high-pressure high-temperature run, the DAC was heated using a Watlow 240 V (rated at 4.65 W·cm^−2^) coiled resistive heater wrapped around it while contained within a custom-built vacuum vessel^43^. The temperature was measured using a K-type thermocouple attached to the gasket. The accuracy of the thermocouple over the temperature range covered by the experiments is ∼0.4%^18,44^ In these experiments, NaCl powder was included in the sample chamber to act as pressure marker^45^. Diffraction patterns were collected at different pressures for 20 s up to 10 GPa. LaB_6_ powder was used for distortion correction, and integration to conventional 2θ-intensity data was carried out with Dioptas software^46^. The indexing and refinement of the powder patterns were performed using the Unitcell^47^, Powdercell^48^ and Fullprof^49^ program packages.

For HP single-crystal XRD measurements we have used a Mini-Bragg DAC from Almax-EasyLab, with an opening angle of 85º and anvil culets of 500 μm diameter, fitted with a stainless steel gasket containing a hole of 200 μm diameter and 70 μm depth. A 4:1 methanol-ethanol mixture was used as pressure-transmitting medium, which assures hydrostaticity up to 10 GPa^41^. The sample was placed on one of the diamonds anvils (diffracted side) together with a small ruby sphere as pressure sensor^50^. The structure was refined, for each pressure, using previous results as a starting point, on *F*^*2*^ by full-matrix least-squares refinement using the SHELXL program^47^. Due to limitations of the opening angle of our DAC, it is only possible to collect about 35% of the reflections present in a full dataset for monoclinic space group at ambient conditions. Numerical absorption correction based on gaussian integration over a multifaceted crystal model was applied using the ABSORB-7 program^51^. After the phase transition, the new structure was resolved using direct methods with the Sir2019 program^52^. All atoms were refined isotropically for HP structures. No restraints were used during this process.

Uncertainties in lattice parameters and atomic coordinates presented in the manuscript come from least-square refinements to our measured data.

### Computational details

The total energies and equations of state of all phases were calculated using density functional theory (DFT) in the periodic plane-wave/pseudopotentials approximation within the Projector Augmented Wave (PAW) formalism^53^, as implemented in Quantum ESPRESSO^54^, version 6.5. The PBEsol exchange–correlation functional was used^55^ and PAW datasets from the pslibrary version 1.0^56^ with 10 (Ba), 10 (Ca), 4 (C), and 6 (O) valence electrons.

After exploring the convergence of the total energy and stress tensor with respect to the calculation parameters, we chose a cutoff energy for the plane wave expansion of 100 Ry, and 1000 Ry for the electron density expansion. Similarly, we chose shifted uniform k-point grids with size 3 × 3 × 3 for all phases except *P*321 alstonite (1 × 1 × 4). These parameters ensure a convergence of about 0.1 mRy in the total energy and around 0.01 GPa in the pressure.

We carried out geometry relaxations at zero and 50 GPa, and then used the calculated equilibrium volumes at those pressures to establish a uniform volume grid with 41 points. At each of those volumes, we performed a constant-volume geometry minimization to find the energy-volume curve and the evolution of the structural parameters as a function of pressure. In all cases, tight relaxation convergence thresholds were used (10^–5^ Ry in the energy and 10^–4^ Ry/bohr in the forces). The resulting energy-volume data was fitted to an analytical strain polynomial expansion using the gibbs2 program^57,58^ and used to obtain the enthalpy-pressure diagram and the phase transition sequence.

## Supplementary Information

Supplementary Information 1.

Supplementary Information 2.

## Data Availability

The crystallographic data of the high-pressure post-barytocalcite BaCa(CO_3_)_2_ polymorph at 9 GPa is deposited and it is publicly available in the Cambridge Structural Database (Deposition number 2145143). The direct link to the deposited data is: (https://www.ccdc.cam.ac.uk/structures/Search?Ccdcid=2145143&DatabaseToSearch=Published). Other data that support the findings are available from the corresponding author on reasonable request.
