# Supplementary material for: Phase stability and dense polymorph of the BaCa(CO3)2 barytocalcite carbonate
Source: Sci Rep. 2022 May 6;12:7413. doi: 10.1038/s41598-022-11301-w (PMC9076881; doi:10.1038/s41598-022-11301-w)

**SUPPLEMENTARY MATERIAL**

**of**

**Phase stability and dense polymorph of the BaCa(CO_3_)_2_ barytocalcite carbonate**

R. Chuliá-Jordán^1^, D. Santamaría-Pérez^1,^*, J. González-Platas^2^, A. Otero-de-la-Roza^3^, J. Ruiz-Fuertes^4^, C. Popescu^5^

*^1^ Departamento de Física Aplicada-ICMUV, Universitat de València, MALTA Consolider Team, 46100 Valencia, Spain*

*^2^ Departamento de Física - Instituto Universitario de Estudios Avanzados en Física Atómica, Molecular y Fotónica (IUDEA), MALTA Consolider Team, Universidad de La Laguna, Avenida Astrofísico Fco. Sánchez s/n, La Laguna, Tenerife E-38204, Spain.*

*^3^ Departamento de Química Física y Analítica, Facultad de Química, Universidad de Oviedo, MALTA Consolider Team, 33006, Oviedo, Spain*

*^4^ DCITIMAC, Universidad de Cantabria, MALTA Consolider Team, 39005 Santander, Spain*

*^5^ CELLS-ALBA Synchrotron Light Facility, Cerdanyola del Vallès 08290, Barcelona, Spain*

**Table 1S**.- Parameters and results of single-crystal XRD data collection, data reduction, and crystal refinement of BaCa(CO_3_)_2_ barytocalcite as a function of pressure.

|  | **Single crystal data** | | | | | | | | | | |
| --- | --- | --- | --- | --- | --- | --- | --- | --- | --- | --- | --- |
| **Pressure (GPa)** | 0.00 | 0.25 | 0.66 | 1.16 | 1.73 | 2.20 | 2.84 | 3.34 | 3.95 | 4.54 | 5.17 |
| **Space Group** | *P*2_1_/*m* | | | | | | | | | | |
| ***a* (Å)** | 6.5503(2) | 6.51(3) | 6.499(16) | 6.467(14) | 6.464(17) | 6.466(12) | 6.451(9) | 6.443(10) | 6.423(13) | 6.45(2) | 6.430(10) |
| ***b* (Å)** | 5.2434(2) | 5.2589(14) | 5.2343(8) | 5.2459(7) | 5.2315(9) | 5.2232(7) | 5.2202(4) | 5.2210(5) | 5.2082(6) | 5.2005(12) | 5.1969(5) |
| ***c* (Å)** | 8.1091(3) | 8.086(6) | 8.050(4) | 8.039(4) | 7.974(4) | 7.941(3) | 7.906(2) | 7.876(3) | 7.830(3) | 7.799(6) | 7.764(3) |
| **β (º)** | 106.019(4) | 106.2(2) | 106.23(14) | 106.79(13) | 106.97(14) | 106.89(10) | 106.78(8) | 107.07(9) | 107.24(12) | 107.4(2) | 107.41(9) |
| **Volume (Å^3^)** | 267.699(17) | 265.8(11) | 262.9(7) | 261.1(6) | 257.9(7) | 256.6(5) | 254.9(4) | 253.3(4) | 250.2(5) | 249.7(10) | 247.5(4) |
| **Z** | 2 | | | | | | | | | | |
| **Density (g/cm^3^)** | 3.690 | 3.716 | 3.757 | 3.784 | 3.830 | 3.849 | 3.875 | 3.900 | 3.949 | 3.957 | 3.991 |
|  | | | | | | | | | | | |
|  | **Crystal structure refinement** | | | | | | | | | | |
| **Nr. reflections** | 717 | 201 | 209 | 208 | 203 | 201 | 206 | 200 | 205 | 197 | 192 |
| **R factor** | 0.0202 | 0.0714 | 0.0280 | 0.0484 | 0.0514 | 0.0332 | 0.0213 | 0.0268 | 0.0262 | 0.0387 | 0.0266 |
| **w_R_ factor** | 0.0417 | 0.1643 | 0.0715 | 0.1287 | 0.1256 | 0.0780 | 0.0531 | 0.0636 | 0.0692 | 0.1018 | 0.0712 |
| **Nr. parameters** | 56 | 27 | 27 | 27 | 27 | 27 | 28 | 28 | 28 | 28 | 28 |
| **GooF** | 1.095 | 1.067 | 1.141 | 1.219 | 1.150 | 1.119 | 1.188 | 1.162 | 1.166 | 1.124 | 1.147 |

**Table 2S.**- Experimentally determined lattice parameters (*a, b, c* and *β*) and unit cell volume of *P*2_1_/*m* BaCa(CO_3_)_2_ barytocalcite at different pressures from powder synchrotron XRD measurements. Pressure uncertainties are estimated to be smaller than 0.05 GPa in the studied range.

| **Pressure (GPa)** | ***a* axis (Å)** | ***b* axis (Å)** | ***c* axis (Å)** | **β angle (º)** | **Volume (Å^3^)** |
| --- | --- | --- | --- | --- | --- |
| 10^-4^ | 6.545(3) | 5.2387(13) | 8.105(3) | 106.09(3) | 267.02(12) |
| 0.05 | 6.544(3) | 5.2371(13) | 8.098(3) | 106.09(3) | 266.64(12) |
| 0.14 | 6.544(2) | 5.2361(13) | 8.094(3) | 106.09(3) | 266.45(12) |
| 0.27 | 6.541(2) | 5.2341(14) | 8.089(3) | 106.10(4) | 266.06(12) |
| 0.41 | 6.537(2) | 5.2338(16) | 8.075(3) | 106.13(4) | 265.39(13) |
| 0.58 | 6.531(2) | 5.2298(14) | 8.055(3) | 106.21(3) | 264.18(13) |
| 0.84 | 6.525(2) | 5.2266(16) | 8.032(3) | 106.27(3) | 262.95(12) |
| 1.15 | 6.517(2) | 5.226(2) | 8.006(3) | 106.36(3) | 261.60(12) |
| 1.49 | 6.501(2) | 5.223(2) | 7.999(3) | 106.41(3) | 260.56(13) |
| 1.92 | 6.491(2) | 5.2189(18) | 7.972(3) | 106.58(3) | 258.82(11) |
| 2.40 | 6.4792(18) | 5.2117(16) | 7.952(3) | 106.73(3) | 257.15(11) |
| 2.78 | 6.4647(18) | 5.2085(16) | 7.921(3) | 106.73(3) | 255.44(11) |
| 3.66 | 6.446(2) | 5.2032(17) | 7.881(3) | 107.09(4) | 252.66(14) |
| 4.40 | 6.434(2) | 5.192(2) | 7.861(3) | 107.30(3) | 250.73(11) |

**Table 3S.**- DFT-calculated lattice parameters (*a, b, c* and *β*) and unit cell volume of *P*2_1_/*m* BaCa(CO_3_)_2_ barytocalcite at different pressures.

| **Pressure (GPa)** | ***a* axis (Å)** | ***b* axis (Å)** | ***c* axis (Å)** | **β angle (º)** | **Volume (Å^3^)** |
| --- | --- | --- | --- | --- | --- |
| 0.015 | 6.552378 | 5.260933 | 8.108442 | 106.3949 | 268.14599 |
| 0.684 | 6.534221 | 5.253270 | 8.062025 | 106.5665 | 265.24982 |
| 1.391 | 6.516475 | 5.246363 | 8.015039 | 106.7766 | 262.35365 |
| 2.140 | 6.499072 | 5.239988 | 7.966373 | 106.9881 | 259.45747 |
| 2.936 | 6.481922 | 5.233412 | 7.918072 | 107.2200 | 256.56130 |
| 3.783 | 6.464493 | 5.227344 | 7.869513 | 107.4674 | 253.66513 |
| 4.684 | 6.447307 | 5.221632 | 7.820350 | 107.7312 | 250.76895 |
| 5.639 | 6.430653 | 5.216083 | 7.770487 | 108.0103 | 247.87278 |

**Table 4S.**- Parameters and results of single-crystal XRD data collection, data reduction, and crystal refinement of HP BaCa(CO_3_)_2_ post-baritocalcite as a function of pressure.

|  | **Single crystal data** | | | | |
| --- | --- | --- | --- | --- | --- |
| **Pressure (GPa)** | 5.9 | 6.4 | 7.0 | 8.0 | 9.0 |
| **Space Group** | *Pm* | | | | |
| ***a’* (Å)** | 6.451(10) | 6.440(10) | 6.420(6) | 6.431(5) | 6.415(5) |
| ***b’* (Å)** | 10.2907(7) | 10.2978(8) | 10.2940(5) | 10.2734(5) | 10.2564(5) |
| ***c’* (Å)** | 7.775(3) | 7.752(4) | 7.731(2) | 7.676(2) | 7.635(2) |
| ***β*’’ (º)** | 110.54(10) | 110.87(11) | 111.15(6) | 111.59(6) | 111.87(6) |
| **Volume (Å^3^)** | 483.3(8) | 480.4(9) | 476.5(5) | 471.5(5) | 466.2(4) |
| **Z** | 4 | | | | |
| **Density (g/cm^3^)** | 4.088 | 4.113 | 4.146 | 4.190 | 4.238 |
|  | | | | | |
|  | **Crystal structure refinement** | | | | |
| **Nr. reflections** | 538 | 552 | 540 | 419 | 594 |
| **R factor** | 0.0268 | 0.0256 | 0.0420 | 0.0238 | 0.0298 |
| **w_R_ factor** | 0.0682 | 0.0668 | 0.1114 | 0.0615 | 0.0792 |
| **Nr. parameters** | 93 | 88 | 93 | 93 | 93 |
| **GooF** | 1.092 | 1.158 | 1.081 | 1.082 | 1.057 |

**Table 5S.**- Experimentally determined lattice parameters (*a’, b’, c’* and *β’*) and unit cell volume of HP *Pm* BaCa(CO_3_)_2_ post-barytocalcite at different pressures from powder synchrotron XRD measurements. Pressure uncertainties are estimated to be smaller than 0.1 GPa in the studied range.

| **Pressure (GPa)** | ***a’* axis (Å)** | ***b’* axis (Å)** | ***c’* axis (Å)** | ***β’* angle (º)** | **Volume (Å^3^)** |
| --- | --- | --- | --- | --- | --- |
| 5.7 | 6.442(4) | 10.310(4) | 7.796(4) | 110.41(8) | 485.2(3) |
| 6.2 | 6.449(4) | 10.305(4) | 7.768(5) | 110.64(8) | 483.1(3) |
| 6.5 | 6.438(5) | 10.315(4) | 7.724(5) | 110.74(9) | 479.7(4) |
| 7.2 | 6.427(5) | 10.309(4) | 7.746(5) | 111.35(9) | 478.0(4) |

**Table 6S.**- DFT-calculated lattice parameters (*a’, b’, c’* and *β’*) and unit cell volume of HP *Pm* BaCa(CO_3_)_2_ post-barytocalcite at different pressures.

| **Pressure (GPa)** | ***a’* axis (Å)** | ***b’* axis (Å)** | ***c‘* axis (Å)** | ***β*’ angle (º)** | **Volume (Å^3^)** |
| --- | --- | --- | --- | --- | --- |
| 5.674 | 6.457389 | 10.26879 | 7.835070 | 109.9185 | 488.4604 |
| 6.543 | 6.446723 | 10.26037 | 7.786375 | 110.3015 | 483.0416 |
| 7.462 | 6.436583 | 10.25166 | 7.737866 | 110.7013 | 477.6227 |
| 8.436 | 6.426641 | 10.24220 | 7.690013 | 111.1120 | 472.2038 |
| 9.468 | 6.416853 | 10.23200 | 7.642724 | 111.5308 | 466.7849 |
| 10.564 | 6.407035 | 10.22091 | 7.596298 | 111.9571 | 461.3660 |

**Figure 1S**.- Arrangements of carbonate [CO_3_] groups in the structures of BaCa(CO_3_)_2_ *P*2_1_/*m* barytocalcite (a) and high-pressure *Pm* post-barytocalcite (b) to illustrate their displacements, tilting and rotations at the phase transition. The Ba and Ca atoms have been removed for more clarity. Note that the *b* axis of high-pressure phase is twice that of the low-pressure barytocalcite. Light gray and red spheres represent C and O atoms, respectively. Cell edges are depicted as solid black lines. Magenta solid lines on the barytocalcite projection demarcate the location of post-barytocalcite unit cell contents, for the sake of comparison between structures. The carbonate group encircled in green has rotated 60º, producing the increase of the coordination number in adjacent Ba and Ca atoms.


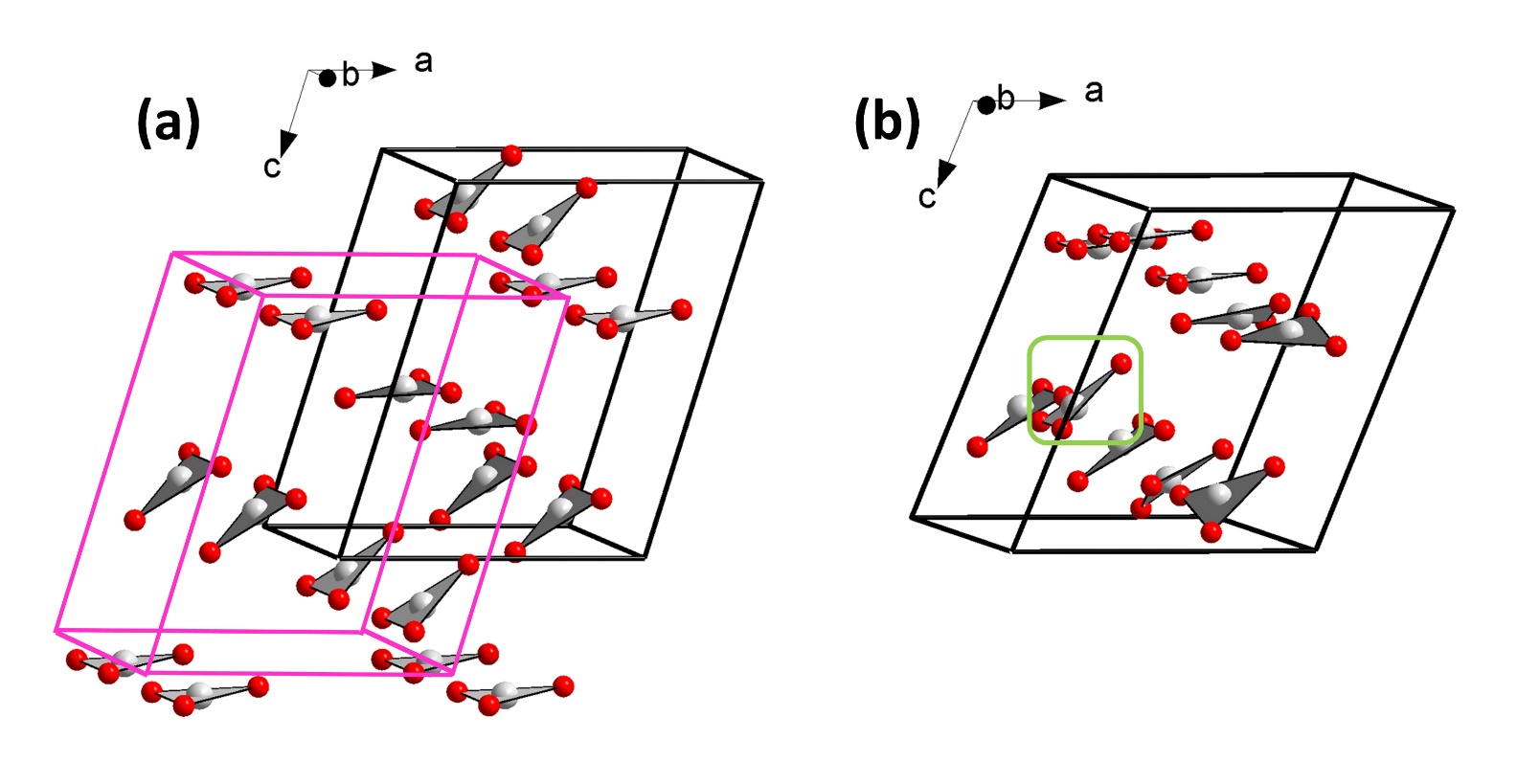

Supplement: Supplementary file 2 — Supplementary Information 2. [file 41598_2022_11301_MOESM2_ESM.docx]
